# Diabetes diminishes muscle precursor cell-mediated microvascular angiogenesis

**DOI:** 10.1371/journal.pone.0289477

**Published:** 2023-08-04

**Authors:** Francisca M. Acosta, Settimio Pacelli, Christopher R. Rathbone

**Affiliations:** 1 Department of Biomedical and Chemical Engineering, University of Texas at San Antonio, San Antonio, TX, United States of America; 2 UTSA-UTHSCSA Joint Graduate Program in Biomedical Engineering, San Antonio, TX, United States of America; 3 Institute of Regenerative Medicine, University of Texas at San Antonio, San Antonio, TX, United States of America; Indiana University Purdue University at Indianapolis, UNITED STATES

## Abstract

The skeletal muscles of Type II diabetic (T2D) patients can be characterized by a reduced vessel density, corresponding to deficiencies in microvascular angiogenesis. Interestingly, T2D also inhibits the function of many myogenic cells resident within skeletal muscle, including satellite cells, which are well-known for the role they play in maintaining homeostasis. The current study was undertaken to gain a better understanding of the mechanisms whereby satellite cell progeny, muscle precursor cells (MPCs), influence microvascular angiogenesis. Network growth and the expression of genes associated with angiogenesis were reduced when microvessels were treated with conditioned media generated by proliferating MPCs isolated from diabetic, as compared to control rat skeletal muscle, a phenomenon that was also observed when myoblasts from control or diabetic human skeletal muscle were used. When only exosomes derived from diabetic or control MPCs were used to treat microvessels, no differences in microvascular growth were observed. An evaluation of the angiogenesis factors in control and diabetic MPCs revealed differences in Leptin, vascular endothelial growth factor (VEGF), IL1-β, interleukin 10, and IP-10, and an evaluation of the MPC secretome revealed differences in interleukin 6, MCP-1, VEGF, and interleukin 4 exist. Angiogenesis was also reduced in tissue-engineered skeletal muscles (TE-SkM) containing microvessels when they were generated from MPCs isolated from diabetic as compared to control skeletal muscle. Lastly, the secretome of injured control, but not diabetic, TE-SkM was able to increase VEGF and increase microvascular angiogenesis. This comprehensive analysis of the interaction between MPCs and microvessels in the context of diabetes points to an area for alleviating the deleterious effects of diabetes on skeletal muscle.

## Introduction

Given the importance of the perfusion of skeletal muscle fibers in maintaining homeostasis, it is intuitive that reductions in capillary density and disruptions in the microvascular function that accompany diseases, like Type II diabetes (T2D), are associated with deleterious consequences in skeletal muscle [1–3]. Satellite cells, resident adult muscle stem cells, are widely recognized for their contribution to muscle homeostasis and muscle repair through their direct participation in myogenesis. The ability of satellite cells to influence their microenvironment through the secretion of factors that impact, for example, neurogenesis, inflammation, and angiogenesis, to name a few, is recognized as an additional satellite cell attribute [4–6]. With regards to the latter, the observation that resident stem cells within skeletal muscle called satellite cells are closely associated with blood vessels *in vivo*, and that the number of satellite cells positively correlates with capillarization implies that an association between satellite cells and microvessels may contribute to muscle homeostasis by affecting the vasculature [7, 8]. Collectively, when specifically taking T2D into consideration, the knowledge that satellite cell content, regenerative capacity, and capillary density are all reduced in muscles in diabetes and/or obesity suggests the interaction between satellite cells and microvessels in diabetic muscle is a critical facet of the disease [9–14].

*In vitro* experiments support the idea that satellite cells exert a positive influence over microvessels. Factors secreted by the progeny of satellite cells (myoblasts, MPCs, MuSCs, etc.) or their immortalized cell equivalent (C2C12 cells) can stimulate angiogenesis [15, 16]. For the remainder of the manuscript, the progeny of satellite cells will be referred to as muscle precursor cells (MPCs) where appropriate. Following in line with the idea that this interaction may be impaired in the context of disease, secretions from muscle cells taken from unhealthy tissue, or treated to resemble diseased cells, have a reduced ability to stimulate angiogenesis [17–20]. The types and/or quantity of growth factors secreted by diseased satellite cells (or their *in vitro* progeny) that are responsible for this insufficiency are beginning to be delineated. For example, myotubes derived from T2D subjects produced an increase in the level of Interleukin 8 that was associated with a reduction in human umbilical vein (HUVEC) tube formation and capillary outgrowth [19]. Conversely, decreases in Vascular Endothelial Growth Factor (VEGF) from aged rat or mouse dystrophic satellite cells were associated with reductions in microvascular angiogenesis [20]. Both the *in vivo* observations alluding to a relationship between satellite cells and microvessels and the *in vitro* studies describing MPC-dependent effects on angiogenesis provide the impetus to gain a better understanding of the mechanisms underlying the interaction between muscle cells and microvessels.

It is becoming increasingly apparent that cell-to-cell communication is affected by the controlled release of exosomes (vesicles ranging from ~50–200 nm) released into the extracellular space carrying RNA, protein, and microRNA (miRNA) to their target cells [21]. When taking into consideration skeletal muscle, there is compelling evidence that satellite cells and their progeny communicate using extracellular vesicles (EVs), including exosomes, to communicate with other myogenic cells, as well as non-myogenic cells; miRNAs contained within them are an important component of the exosome cargo through which the signals are realized [6, 22–25]. For example, Fry et al. [23] demonstrated that muscle precursor cells secrete miR-206, which effectively downregulates collagen biosynthesis in fibrogenic cells to support muscle hypertrophy. With regards to angiogenesis, exosomes derived from healthy C2C12 cells stimulate HUVEC angiogenesis, however, oxidative stress negatively regulates exosome-induced angiogenesis, supporting the idea that disease negatively alters the exosomal profile of satellite cells [16, 18].

Collectively, a thorough understanding of the impact of disease on both MPC derived growth factors and EVs, and their ability to influence angiogenesis is warranted. In the current study, microvascular fragments (MVFs) were used as a means to determine whether a change in the factors (using conditioned media or exosomes) secreted by proliferating diabetic MPCs can contribute to reduced vascular integrity. In addition, the interaction between diseased myogenic cells and microvessels was explored in a tissue-engineered skeletal muscle (TE-SkM) model where myotubes derived from healthy and diseased MPCs, and MVFs, could be evaluated in a three-dimensional configuration. Finally, the effect of injury on the secretome of diseased cells was evaluated as the effect of MVF angiogenesis was evaluated after treatment from conditioned media procured from injured TE-SkMs. An analysis of the differences in the healthy and diabetic MPC secretomes identified several factors that may be involved in the diminished ability of diseased cells to influence angiogenesis. This comprehensive analysis involving MPCs and microvessels provides additional insight into insufficiencies associated with vascularization in diabetic skeletal muscle.

## Materials and methods

### Animals

Muscle precursor cells (MPCs) were isolated from control (FA/+) or obese (FA/FA) male Zucker diabetic fatty (ZDF) rats obtained from Charles River (Wilmington, MA). Microvascular fragments (MVFs) were isolated from adult male Lewis rats (Envigo, Indianapolis, IN). ZDF rats were obtained at 4 weeks of age and fed Purina 5008 until 15–16 weeks old, similar to that described previously [26, 27]. All animals were housed in a temperature-controlled environment with a 12-h light-dark cycle and fed *ad libitum*. This study was conducted in compliance with the Animal Welfare Act and the Implementing Animal Welfare Regulations, in accordance with the principles of the Guide for the Care and Use of Laboratory Animals, and was approved by the Institutional Animal Care and Use Committee at the University of Texas at San Antonio.

### Muscle precursor cell isolation and culture

Rat muscle precursor cells (MPCs) were isolated similar to that described previously [28]. Briefly, skeletal muscles (tibialis anterior, extensor digitorum longus, quadriceps, gastrocnemius, and plantaris) were collected from each rat, minced, digested with protease type IV, and filtered with a 100 μm Steriflip to remove debris. Harvested cells were plated on a tissue culture-treated plate for 12 hours. Subsequently, non-adherent MPCs were transferred to a Matrigel-coated dishes, and MPCs were then cultured in growth medium (GM) composed of 20% fetal bovine serum (FBS), 1% penicillin-streptomycin (P/S), 0.2% MycoZap (MZ), Dulbecco’s Modified Eagle Medium (DMEM) supplemented with 1 g/L D-glucose, L-glutamine, and 110 mg/L sodium pyruvate. Human MPCs (HSMM; muscle myoblasts (CC-2580) or D-HSMM (CC2901)) were purchased, (Lonza, Basel, Switzerland) and cultured similarly to that described for rat MPCs.

### Conditioned media exosome isolation and characterization

MPCs (passage 2) from control and diabetic rats were cultured on Matrigel-coated 150-mm dishes until ~80% confluency before passaging for complete media (CM) and exosome (Exo) collection. Briefly, prior to CM collection from proliferating MPCs, plates were washed twice with PBS and then cultured in DMEM containing 1% P/S and 0.2% MZ without FBS for 24 hours. Similarly, for TE-SkM after maturation, constructs were washed twice with PBS and then cultured in DMEM containing 1% P/S and 0.2% MZ without FBS for 24 hours. The CM was then removed and centrifuged for 10 minutes at 300 g to remove any cellular debris and stored at 4°C before being analyzed or used for MVF treatments. To obtain Exo, a fraction of the CM was subjected to centrifugation at 2,000 g for 10 minutes, followed by another step of centrifugation at 10,000 g for 1 hour. The supernatant was then filtered through a 0.22 μm filter and transferred to Ultra-Clear centrifuge Tubes (Beckman Coulter) before being ultracentrifuged twice for 1 hour and 20 minutes at 10,000 g. The pellet containing exosomes was resuspended in 200 μL of PBS pH 7.4 and used immediately for further experiments or frozen at -80°C for future analyses.

After lysis in RIPA buffer (89900, ThermoFisher Scientific, Waltham, MA) containing protease and phosphatase inhibitors (PPC1010, Sigma-Aldrich, St. Louis, MO), the protein contents of CM and Exo were determined using a BCA assay (EMD Millipore, Burlington, MA). For both CM and Exo, the determination of protein content allowed for the addition of equal amounts of protein across groups (e.g., control vs diabetic) within a treatment (e.g., CM or Exo). To verify the presence of exosomes, Exo lysates were subjected to western blot analyses for CD63 or TSG101.

### Insulin-stimulated glucose uptake (ISGU)

Insulin-stimulated glucose analysis was carried out on MPCs according to the manufacturer’s instructions (Glucose Uptake-Glo^TM^ Assay, Promega, Madison, WI). Briefly, MPCs were grown in 96-well plates until ~80% confluency and then cultured in DMEM without serum or glucose for 24 hours. Samples were changed to DMEM ± insulin (1mM) for 1 hour at 37°C, followed by the removal of DMEM ± insulin and the addition of 2-Deoxyglucose (0.1mM) in PBS for 30 minutes at room temperature. Finally, a 2-Deoxyglucose-6-phosphate (2DG6P) detection reagent was used to quantify the amount of glucose internalized by the cells. Luminescence was measured after 1 hour at room temperature with a spectrophotometer (Biotek, Vinooski, VT).

### Microvascular fragment isolation and hydrogel formation

Microvascular fragments (MVFs) were isolated from the epididymal, inguinal, and subcutaneous fat depots in a similar manner described in previous studies [29, 30]. Based on the differences among angiogenesis between MVFs derived from epididymal, inguinal, and subcutaneous fat depots, only male rats were used as a source of MVFs and MPCs, i.e., MVF based assays where the epididymal fat pad was excluded (female rats) were not used. Briefly, adipose tissue from the epididymal (EPI), inguinal (ING), or subcutaneous (SUBQ) fat was subjected to an 8–15 minute collagenase type I digestion (Worthington Biochemical Corporation, Lakewood, NJ) at 37˚C with agitation. The digested material was centrifuged (400g × 4 min), which resulted in a floating layer of adipocytes and a pellet containing a heterogeneous mixture of cells and MVFs. The pellet was resuspended in phosphate-buffered saline (PBS) containing 0.1% bovine serum albumin (Sigma-Aldrich; St. Louis, MO) and filtered through 500 μm and 37 μm filters (Carolina Biological Supply, Burlington, NC) to remove large debris and minimize cell contamination, respectively. MVFs were then counted, combined, centrifuged, and resuspended in fibrinogen (Sigma-Aldrich; St. Louis, Mo.) in DMEM (20 mg/mL) at a concentration of 20,000 MVFs/mL. Fibrin hydrogels (5.7 mg/mL) were formed by combining MVFs containing fibrinogen and 10U/mL thrombin (MilliporeSigma, St. Louis, MO) in 96-well culture plates. Hydrogels for PCR were 100μL in volume (allotting for enough RNA extraction), while gels for all other analyses were 50μL.

### mRNA gene expression analysis

RNA was isolated and purified from hydrogels (100μL) containing MVFs using a Qiagen RNeasy Mini Kit (Valencia, CA) according to the manufacturer’s guidelines. mRNA concentrations were measured using a Take3 Micro-Volume Plate (BioTek, Winooski, VT), then normalized to 150 ng of mRNA for its conversion to cDNA. Isolated RNA was converted to cDNA using the iScript cDNA synthesis kit (Bio-Rad, Hercules, CA). Real-time quantitative polymerase chain reaction (qPCR) was performed using a CFX96 Touch Real-Time PCR Detection System (Bio-Rad, Hercules, CA). All primers used to carry out the qPCR analysis were predesigned primers (Sigma-Aldrich; St. Louis, Mo) listed in Table 1. 10μL of iTaq Universal SYBR Green Supermix (Bio-Rad, Hercules, CA) was used for each reaction. Fold expression levels were calculated using the 2^-ΔΔCt^ method, where the GM gels at day 1 were designated as the calibrator group, and GAPDH expression was used as the housekeeping gene [31].

**Table 1:** PCR primers.

Target Gene	Sequence (5’-3’)
rGAPDH	F – AGCCCAGAACACCATTCCTAC R – ATGCCTGCTTCACCACATTC
rFLK1	F – ACTCACAGTTCCCAGAGTGGTT R – GAATGGTGACCTGTGATCTTGA
rANGPT-1	F – TATTTTGTGATTCTGGTGATT R – TCGCTTTATTTTTGTAATG
rVEGF	F – CAACTTCTGGGCTCTTCTCT R – CTCACCCGTCCATGAGC

**Abbreviations:** r, Rat; F, forward primer; R, reverse primer

### Cytokine multiplex array

Cytokine measurement of cell lysates and culture media derived from control and diabetic rat MPCs was performed using the MILLIPLEX MAP Rat Cytokine/Chemokine Magnetic Bead Panel–Premixed 27 Plex–Immunology Multiplex Assay (Millipore Sigma, St Louis, MO) according to the manufacturer’s instructions. Briefly, control and diabetic MPCs were lysed using Cell lytic M (Sigma Aldrich, St. Louis, MO) for 15 minutes at room temperature. The cell lysates were centrifuged at 12,000 g for 15 minutes to remove all cell debris. The cellular protein contents were determined with a Bradford Assay (Sigma Aldrich, St. Louis, MO), and the samples were then frozen at -20°C at equal concentrations. Similarly, the culture media from control and diabetic cells was centrifuged for 10 minutes at 300 g to remove any cellular debris, followed by centrifugation at 2,000 g for 10 minutes. Additionally, the supernatant was filtered with a 0.22 μm filter, and the protein concentration was determined as described above. All the samples were diluted to 200 μg/mL. 25 μL of cell lysate or conditioned media was used for the assay, and the quantification was carried out according to the manufacturer’s protocol. Briefly, 200 μL Assay buffer was mixed with 25 μL of sample and beads provided in the kit. The mixture was left to incubate for 2 hours at room temperature, the wells were washed with 200 μL of wash buffer followed by incubation for 1 hour with a 25 μL antibody detection mixture and then, 25 μL of Streptavidin-Phycoerythrin was added to each well for 30 minutes at room temperature. The wells were then washed twice with wash buffer, and finally, 125 μL of Sheath Fluid PLUS was added to each well, and cytokines were quantified using Luminex 200 (Luminex Corporation, Austin, TX).

### Tissue-engineered skeletal muscle (TE-SkM) formation and injury

TE-SkM containing MVFs were created similar to that described previously [26, 32]. MPCs from either control or diabetic rats (1.25x10^6^ cells/mL) were resuspended in fibrinogen previously solubilized in DMEM at the concentration of 20 mg/mL with the addition of MVFs at a concentration of 20,000 MVFs/mL. Fibrin gels (5.7 mg/mL) were formed by mixing the cells resuspended in a fibrinogen solution with 10 U/mL of thrombin. The Fibrin gel/cell suspensions (300 μL) were seeded onto polydimethylsiloxane (PDMS) casts with a half-pipe structure containing 2 small metal pins that served as anchor points at each end, so that the final dimensions of the construct were 25mm in length, 4.7mm width, and 2.35mm height. PDMS molds were coated with Pluronic acid (10 mg/mL in PBS) for 48 hours prior to seeding to inhibit the subsequent attachment of the fibrin hydrogels, which was removed and followed by PBS washes prior to gel seeding. Constructs were grown in GM for 4 days before being switched to Differentiation Media (2% horse serum, 1% P/S, 0.2% MZ, and DMEM) for the duration of culture (unless CM was collected). All media throughout the study were supplemented with 1mg/mL aminocaproic acid to prevent fibrinolysis. Under all culture conditions, the appropriate volume of medium (500 μL) was replaced every other day throughout the study, while constructs were maintained in a humidified incubator at 37°C and 5% CO_2_ [26, 32].

To induce injury, a weight of 1 Kg was applied to the samples for 30 seconds. Media was collected from intact and injured constructs 24 hours post-injury, the amount of VEGF was measured using an ELISA (RRV00, R&D Systems, Minneapolis, MN), and then CM was normalized based on the protein concentration. The media was then used to treat MVFs in fibrin gels for 7 days, after which MVF sprouting and growth was determined using Lectin staining, as previously described [30], as well as qPCR analysis of key angiogenic genes. Finally, fluorescence staining of F-actin and DAPI was done on injured/non-injured TE-SkM as described above to visualize the area of injury.

### Evaluation of angiogenesis in TE-SkM

Following 2, 4, and 6 days of culture hydrogels were fixed in 4% formaldehyde for 2 hours at room temperature, permeabilized using 0.5% Triton-X for 20 min, blocked using 10% goat serum for 2 hours, then stained using Rhodamine labeled Griffonia (Bandeiraea) Simplicifolia Lectin I (GS-1; Vector Labs, Burlingame, CA, 1:100) overnight at 4°C, then counter-stained with F-Actin (ThermoFisher, R37110, Waltham, MA) and DAPI (ThermoFisher, R37606, Waltham, MA). Three regions of interest (ROIs) (comprising ~50% of total construct length) per sample were imaged on a Leica TCS SP8 Confocal Microscope (Buffalo Grove, IL) using a rendering of 50 μm thickness/5μm sections. Lectin quantification was performed using the Leica 3D analysis toolkit using Otsu thresholding.

### MVF angiogenesis assay

After an initial culture in GM for 24 hours, MVFs were treated with CM or exosomes derived from control or diabetic MPCs, or CM from control or injured TE-SkM, with the different treatments (100 μL) being replaced every other day throughout the study. All media used for MVF culture was supplemented with 1 mg/mL aminocaproic acid to prevent fibrinolysis. MVFs were grown in conditioned media containing 1% FBS (Pre-CM(-)) without any MPC-derived secretome and served as a negative control, while media containing 20% of FBS (GM(+)) was used as a positive control.

MVF angiogenesis was evaluated using two different methods. For the first method, the hydrogels containing MVFs (50 μL) were fixed in 4% formaldehyde for 2 hours at room temperature after 10 days of culture. Samples were permeabilized using 0.5% Triton-X for 20 min, blocked using 10% goat serum for 2 hours, and stained using Rhodamine labeled Griffonia (Bandeiraea) Simplicifolia Lectin I (GS-1; Vector Labs, Burlingame, CA, 1:100) overnight at 4°C. The distribution of vessels of the entire wells was determined using a Leica TCS SP8 Confocal Microscope (Buffalo Grove, IL). The stacked image (100 μm) of each well containing the MVF within the fibrin hydrogel was obtained by combining 10 images with a thickness of 10 μm. Quantification was performed using the Leica 3D analysis toolkit using Otsu thresholding.

For the second method, a machine learning analysis was used (BioSegment, Advanced Solutions, Louisville, KY) [33]. The software was trained to recognize vessels by manually tracing more than 50 phase-contrast 100X images prior to the analysis. On days 6, 8, and 10, five different images per group were evaluated.

### Statistical analysis

Graphpad Prism Software 9 (GraphPad Software, Inc., La Jolla, CA) was used to run a one-way analysis of variance (ANOVA) tests with Tukey’s multiple comparison analyses or t-tests to determine differences between groups. Statistical significance was determined when *p* < 0.05. All results are presented as mean ± standard error of the mean (SEM). Experiments were performed on separate occasions with at least 3 replicates per experimental group.

## Results

### Comparison between the angiogenic potential of secretome derived from control and diabetic rat MPCs

**Fig 1:**
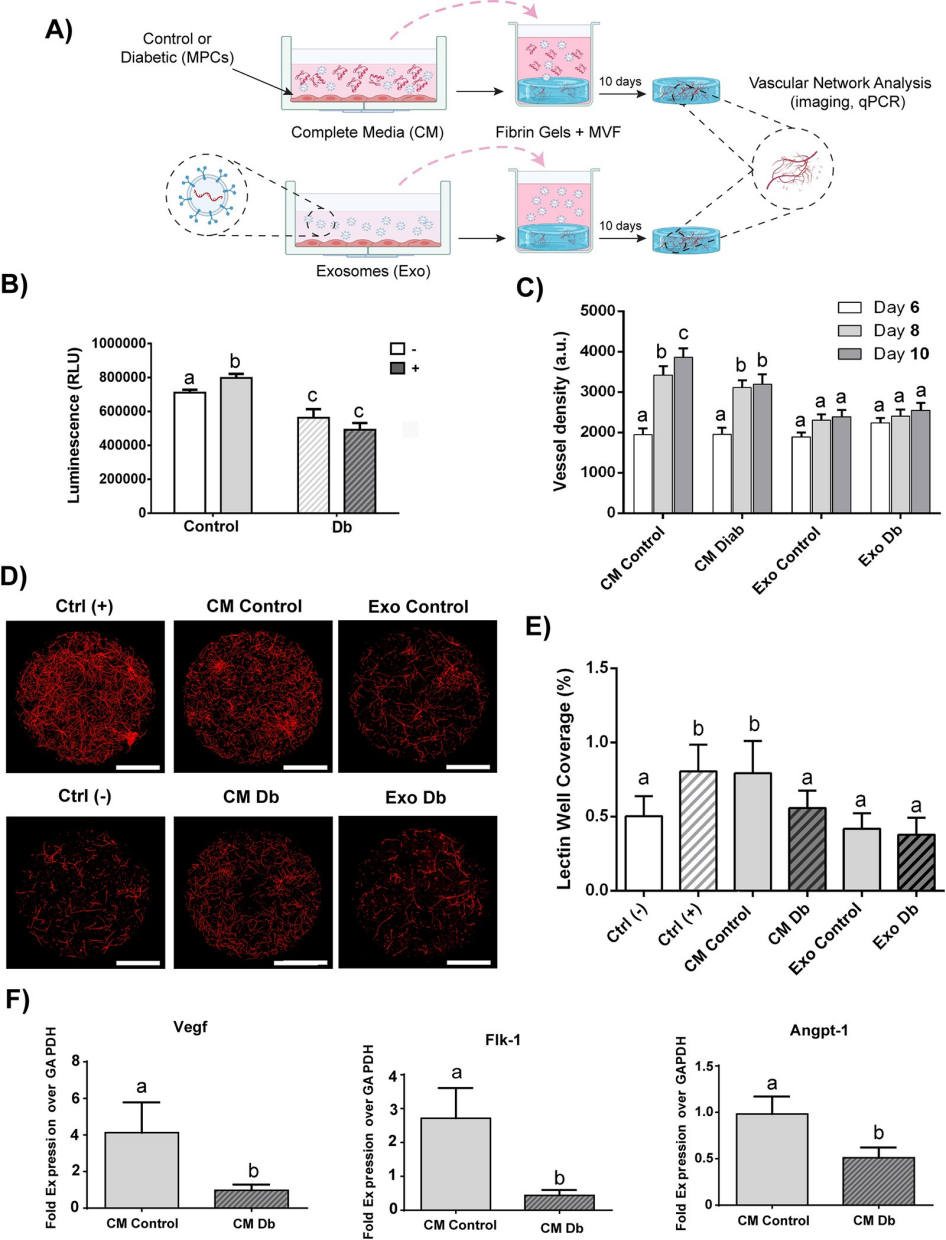
Angiogenesis analyses using secretome sourced from rat MPCs. A) Schematic representing the study design (Created with BioRender.com). MPCs derived from control (+/FA) and diabetic (FA/FA) rats were cultured for 24 hours in serum free media and the secretome (CM or exosomes) collected and used to treat microvascular fragments (MVF). B) Quantification of insulin-stimulated glucose uptake for MPCs derived from control (+/FA) and diabetic (FA/FA) rats. C) Quantification of MVF vessel length over time up to 10 days using Biosegment software. D) Confocal microscopy images of MVFs grown in fibrin hydrogels (plugs) and stained with GS Lectin I (red) to visualize the vessel formation of MVFs cultured for 10 days in the different MPC sourced secretome as well exosomes derived from control and diabetic MPCs. (Scale bar = 500 μm). E) Quantification of vessel formation as a measurement of % well coverage of confocal images for MVFs grown in the different conditions. F) Fold expression of vascular endothelial growth factor (VEGF), Vascular endothelial growth factor receptor 2 gene (Flk-1), and angiopoietin 1 (ANGPT-1) in MVFs. Results were normalized based on their respective day 0 control group. Results are reported as mean ± standard error (n = 6-8/group, values are averages of measurements of at least 2 replicates (wells) derived from each biological replicate (cells derived from 3 separate animals per condition). The presence of different letters represents statistical significance among the groups (p < 0.05). Bars displaying the same letter indicate no statistical significance among the groups.

Conditioned media (CM) derived from MPCs from control (r-Control) and diabetic (r-Db) Zucker rats were collected and used to culture microvascular fragments (MVFs) suspended within fibrin hydrogels. The set-up of the experiments is reported in the schematic (Fig 1A). The amount of glucose taken up by the cells upon insulin stimulation was assessed; r-Control MPCs displayed a significant increase (~14%) in insulin-stimulated glucose uptake (ISGU), which was not observed in the r-Db MPCs (Fig 1B), confirming the diseased phenotype, similar to that described before [26, 27]. MVF exposed to the CM derived from the r-Db MPC group displayed a significant reduction in vascular network formation with ~16–27% reductions in vessel length and histological analyses of the vessel well coverage, when CM from Db cells was used to treat MVFs as compared to CM from control cells (Fig 1C–1E). Notably, no significant difference was seen in either method of vessel formation analysis between r-Control and r-Db when exosomes from either cell type were used to treat MVFs. Vascular endothelial growth factor receptor 2 gene expression (FLK1) in MVFs was reduced by 84% when treated with CM from the r-Db group as compared to the r-Control group (Fig 1F). Similarly, angiopoietin 1 gene expression (ANGPT-1) in MVFs was reduced by 48% when treated with CM from the r-Db group as compared to the r-Control group (Fig 1F). Lastly, vascular endothelial growth factor (VEGF) gene expression in MVFs was reduced by 77% when treated with CM from Db MPCs as compared to treatment with r-Control CM (Fig 1F).

### Cellular and secretome analyses to determine differences between control and diabetic rat MPCs

**Fig 2:**
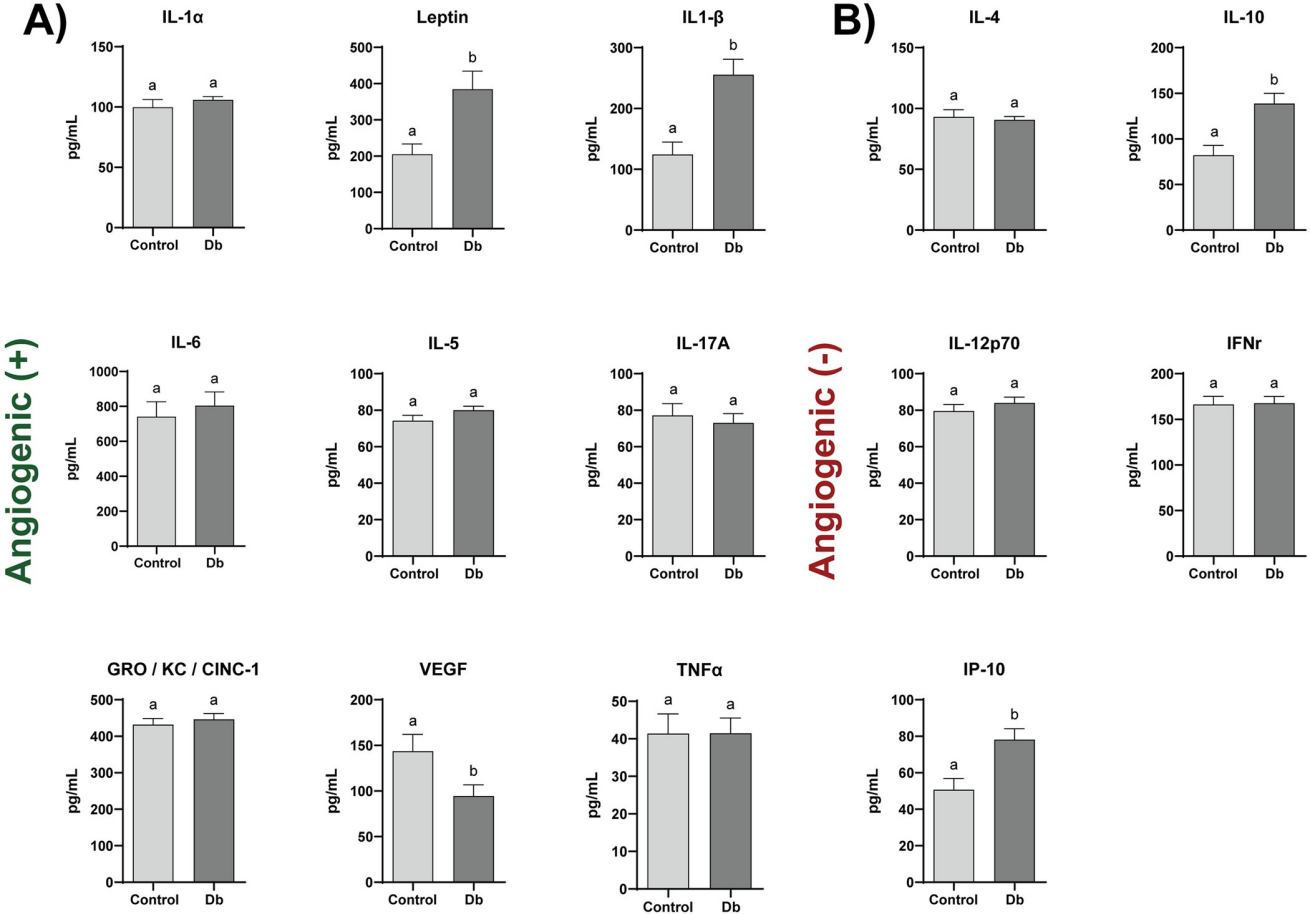
Angiogenic cytokine multiplex array analysis of cell lysates derived from control (+/FA) (r-Control), and diabetic (r-Db) (FA/FA) MPCs. Separated by those that A) promote (+) or B) inhibit (-) angiogenesis. All the results are reported as mean ± standard error (n = 6-9/group, Values are averages of measurements of at least 2 replicates (wells) derived from each biological replicate (cells derived from 3 separate animals per condition)). The presence of different letters represents statistical significance among the groups (p < 0.05). Bars displaying the same letter indicate no statistical significance among the groups.

**Fig 3:**
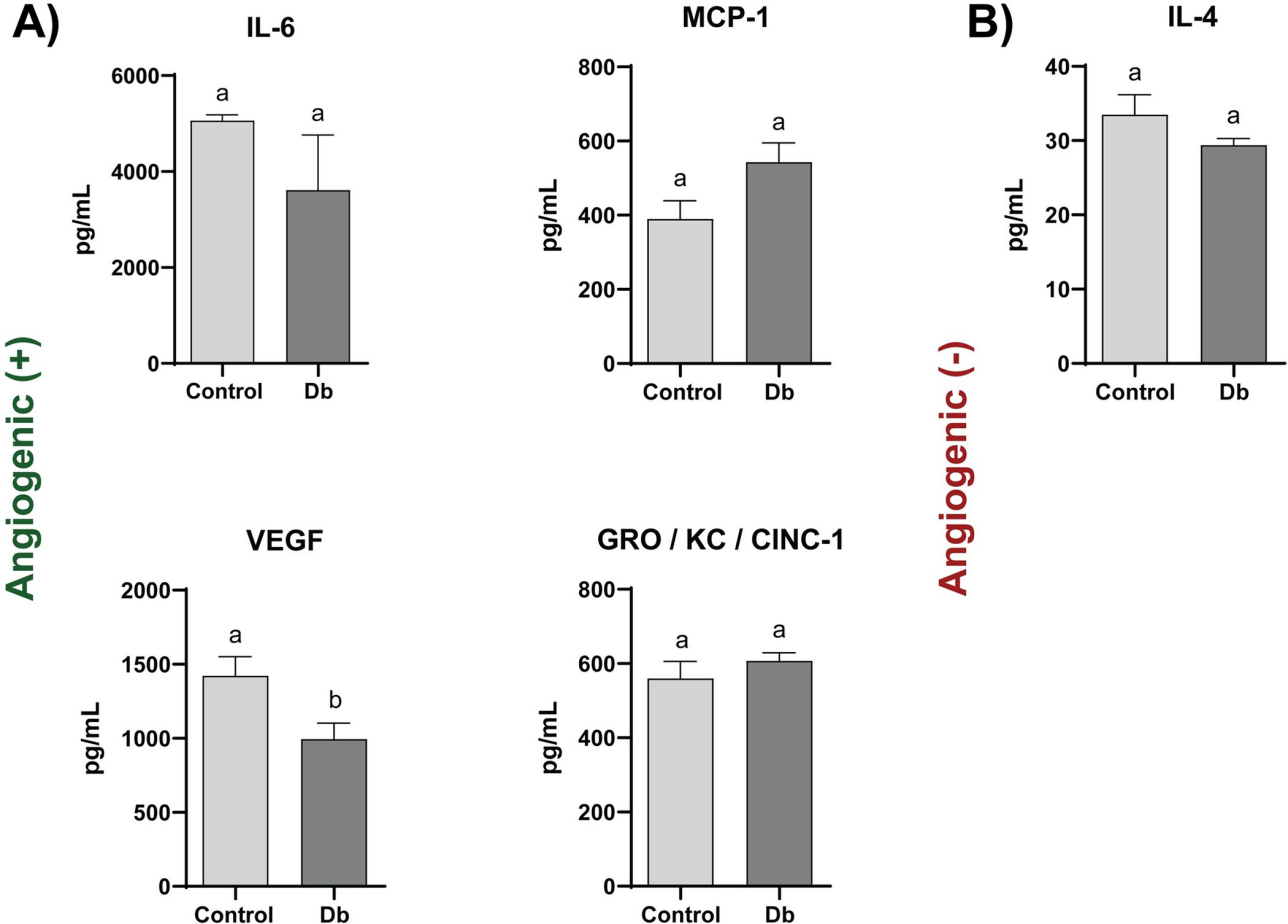
Angiogenic cytokine multiplex array analysis of culture media containing secretome derived from control (+/FA) (r-Control), and diabetic (r-Db) (FA/FA) MPCs. Separated by those that A) promote (+) or B) inhibit (-) angiogenesis. All the results are reported as mean ± standard error (n = 3-6/group, Values are averages of measurements of 1–2 replicates (wells) derived from each biological replicate (cells derived from 3 separate animals per condition)). The presence of different letters represents statistical significance among the groups (p < 0.05). Bars displaying the same letter indicate no statistical significance among the groups.

The cellular contents and composition of the conditioned media (CM) of both control (r-Control) (+/FA) and diabetic (r-Db) (FA/FA) MPCs was thoroughly investigated to draw plausible justifications behind the differences observed in angiogenesis. Specifically, the cell lysates of r-Control and r-Db MPCs were collected and analyzed for genes associated with the promotion of angiogenesis (Fig 2A) and those associated with diminished angiogenesis (Fig 2B). Of the proteins analyzed, Leptin, IL1β, VEGF, IL-10, and IP-10 contents were found to be different between r-Control and r-Db MPCs. The CM from r-Control MPCs and r-Db MPCs was also analyzed for genes associated with the promotion of angiogenesis (Fig 3A) and those related to diminished angiogenesis (Fig 3B). Of the proteins analyzed, VEGF was the only factor that was different between the r-Control and r-Db conditioned media.

### Comparison between the angiogenic potential of human MPCs’ secretome derived from control and diabetic MPCs

**Fig 4:**
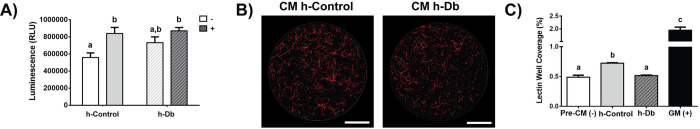
Angiogenesis analyses using secretome sourced from human MPCs. The test groups consisted of MVFs cultured in pre-CM conditions, exposed to secretome derived from human control MPCs (h-Control), or diabetic MPCs (h-Db), or treated with growth media (GM+). **A)** Quantification of insulin-stimulated glucose uptake for human control and diabetic-derived MPCs. Insulin-stimulated cells are indicated in the graph as (+) and the unstimulated cells as (-). **B)** Confocal microscopy images of MVFs grown in fibrin hydrogels (plugs) and stained with GS Lectin I (red) after 10 days. (scale bar = 1 mm). **C)** Quantification of MVFs grown in the different conditioned media by measuring the fluorescence intensity of lectin staining expressed as lectin-well coverage (%). All the results are reported as mean ± standard error (values are averages of measurements of 6–8 replicates (wells) derived from purchased cells derived from one biological replicate (1 subject)). The presence of different letters represents statistical significance among the groups (p < 0.05). Bars displaying the same letter indicate no statistical significance among the groups.

Analogous to the experiments with r-Control and r-Db MPCs, whether a decline in angiogenesis could also be observed in diabetic muscle precursor cells (MPCs) derived from a human source was evaluated. Specifically, myoblasts were purchased, and experiments similar to that described above were carried out, i.e.. conditioned media (CM) derived from control (h-Control) and diabetic (h-Db) human MPCs was collected and used to treat microvascular fragments (MVFs). Corresponding to that observed with rat MPCs, a ~45% increase in insulin-stimulated glucose uptake (ISGU) was observed with control human MPCs, while their diabetic counterparts did not exhibit an increase in ISGU (Fig 4A).

Additionally, both qualitative and quantitative histology was performed on all MVFs grown in fibrin gels and treated with conditioned media taken from healthy or diseased MPCs for 10 days. MVF growth was clearly stunted in CM containing h-Db MPC derived secretome, with a noticeable drop in vessel sprouting and network formation (Fig 4B). Similar to that described with CM derived from rat MPCs, a decrease in angiogenesis was displayed from h-Control to h-Db conditions, with only the h-Control CM group showing a significant increase relative to the negative control by 47% (Fig 4C). Collectively these results, coupled with the results from our rat studies, indicate that the secretome derived from diabetic MPCs seems to possess a decreased pro-angiogenic potential compared to the control MPC group, with both human and rat-derived MPCs having a similar outcome.

### Assessment of the angiogenic response of MVFs in a 3D TE-SkM model containing control and diabetic MPCs

**Fig 5:**
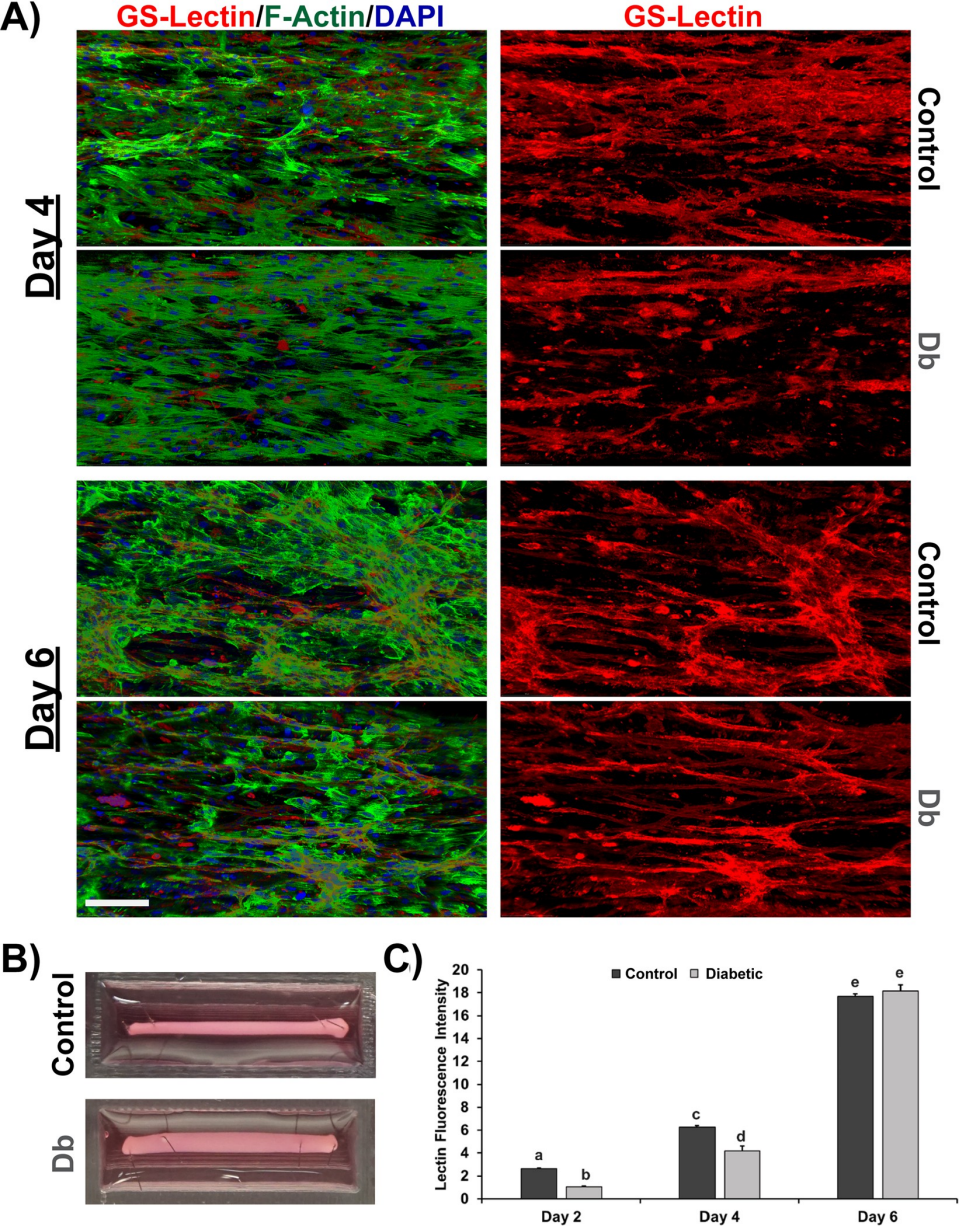
Assessment of MVF growth in TE-SkM constructs co-cultured with MPCs derived from control (+/FA) and diabetic (FA/FA) rats. A) Confocal microscopy images of MVFs grown in TE-SkM and imaged 4 and 6 days post-seeding and stained with GS Lectin I (red) to visualize the vessel formation of MVFs. Scale bar = 200 μm B) Representative images of the construct after six days of culture for both the control and diabetic groups. C) Quantification of vessel formation using Lectin staining. Results are reported as mean ± standard error (n = 6-8/group, values are averages of measurements of at least 2 replicates (constructs) derived from each biological replicate (constructs were made from MPCs derived from 3 separate animals per condition)). The presence of different letters represents statistical significance among the groups (p < 0.05). Bars displaying the same letter indicate no statistical significance among the groups.

After six days of culture, the TE-SkM derived from r-Control MPCs was more compacted than the r-Db group, which is indicative of an increase in maturation, as described previously (Fig 5B) [26]. MVFs’ growth and vascular network organization were investigated by evaluating vessel coverage in confocal images at three different time points (Fig 5A, *Only D4 & 6 Shown*). Interestingly, the growth rate of MVF was faster for the control TE-SkM compared to the diabetic group, as demonstrated by quantification of MVF vessel coverage at 2 and 4 days (Fig 5C).

### Assessment of the angiogenic response of injured TE-SkM model containing control and diabetic MPCs

**Fig 6:**
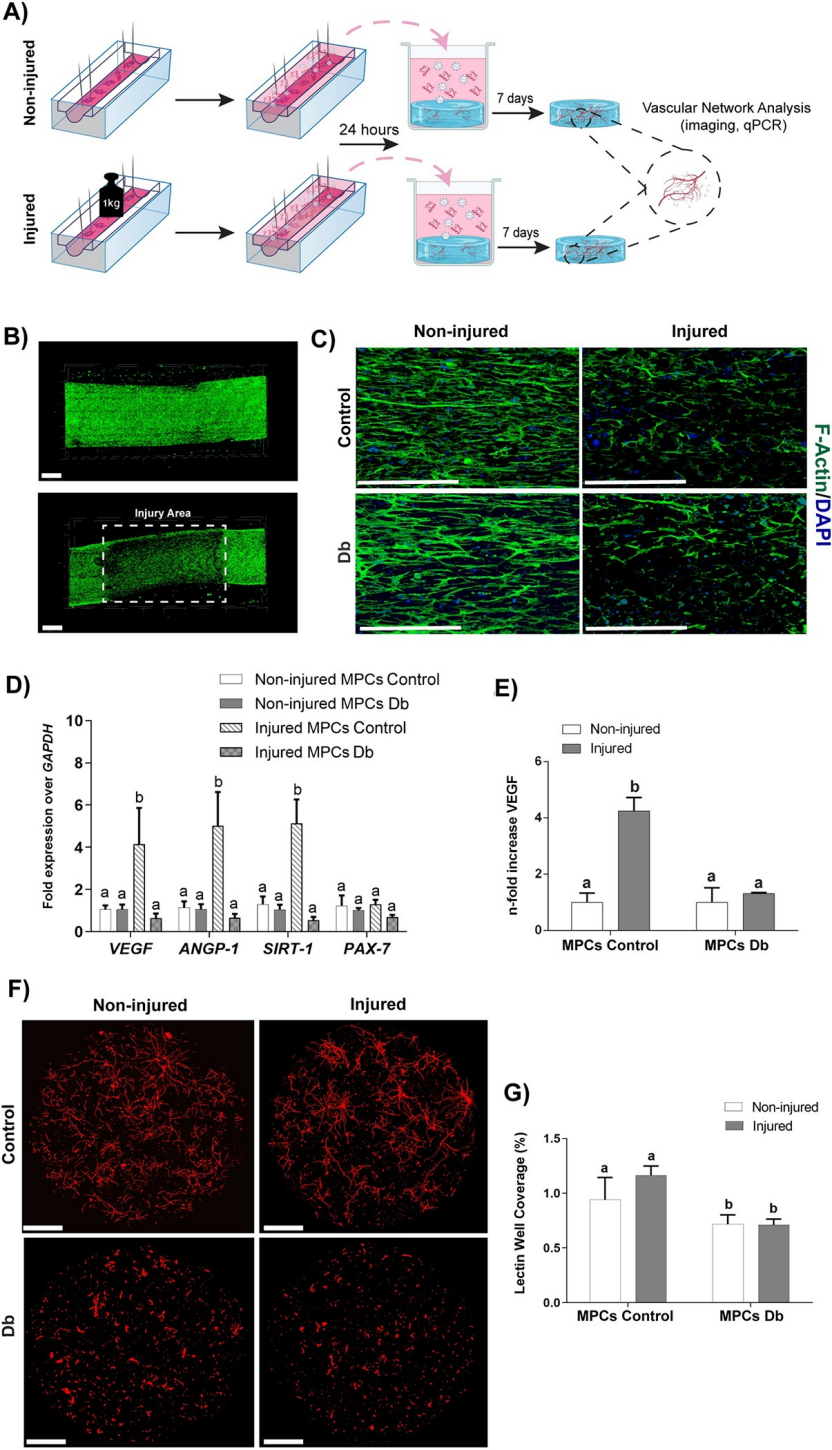
Evaluation of angiogenesis of control and diabetic TE-SkM constructs upon injury. A) Schematic representing the study design. MPCs derived from either control (+/FA) or diabetic (FA/FA) rats were used to develop TE-SkM, which was non-injured/injured and 24 hours later secretome collected and tested on microvascular fragments (MVF) cultured in fibrin hydrogels. MVF growth, vascular density, and angiogenic expression were quantified after 7 days of culture (Created with BioRender.com). B) Representative confocal fluorescent image of TE-SkM stained with actin to identify the injury area (Scale bar = 1 mm). C) Fluorescent images of actin and DAPI stained TE-SkM constructs showing the allignment of control and diabetic MPCs along the axis of the constructs and the disruption in their morphology caused by compression. (Scale bars = 200 μm). D) qPCR analysis of angiogenic genes 24 hours after injury in TE-SkMs made from different MPC sources. E) Quantification of VEGF found in the culture media of TE-SkMs before and after the injury. F) MVF lectin staining cultured in fibrin gels using conditioned media obtained from control and diabetic TE-SkM constructs injured and not injured. (Scale bar = 500 μm). G) Corresponding quantification of vessel formation as a measurement of % well coverage for MVFs grown in the different conditions. Results are reported as mean ± standard error (n = 4-8/group, values are averages of measurements of replicates (wells or constructs) derived from each biological replicate (constructs made from MPCs derived from 3 separate animals per condition)). The presence of different letters represents statistical significance among the groups (p < 0.05). Bars displaying the same letter indicate no statistical significance among the groups.

TE-SkM developed from either control or diabetic MPCs, were subjected to injury, and the CM collected from non-injured and injured constructs and used to treat MVFs similar to that described above (Fig 6A). MPCs were stained with F-actin to visualize the distribution of the cells within the construct and identification of the injured area (Fig 6B). After injury, the alignment of the myogenic structures within the TE-SkM was reduced, appearing highly disorganized (Fig 6C). Control TE-SkM constructs displayed a significant upregulation of two key angiogenic genes, VEGF and Angiopoietin-1 (ANGP-1). Similarly, Sirtuin-1 (SIRT-1), was only upregulated after injury in the healthy TE-SkM constructs. The expression of PAX-7 did not decrease upon injury in both types of constructs (Fig 6D). Secretome analysis of vascular endothelial growth factor (VEGF) revealed a significant increase in its content after one 1-day post-injury only in the TE-SkM derived from MPCs taken from control rats. On the contrary, VEGF did not increase in the constructs derived from MPCs taken from diabetic animals, suggesting a diminished angiogenic response to mechanical injury when diabetic animals were used as the source of MPCs (Fig 6E). Angiogenic potency was further confirmed by examining the sprouting of MVF in fibrin hydrogels cultured using the media obtained from injured and intact TE-SkM constructs (Fig 6F). Angiogenesis was reduced when CM was taken from Db TE-SkM as compared to CM taken from control TE-SkM. (Fig 6G).

## Discussion

Skeletal muscle myonuclei undergo apoptosis as a part of normal skeletal muscle fiber turnover, and the replenishment of myonuclei via myogenesis is largely dependent on resident stem cells in muscle called “satellite cells” [34, 35]. Previous work demonstrating that progeny of satellite cells (i.e., muscle precursor cells) taken from diabetic muscle, and the myotubes that develop from them, exhibit impaired insulin signaling and recapitulate some of the features of the disease were instrumental in gaining a better understanding of the pathology [36–42]. In the current studies, MPCs had a reduction in insulin-stimulated glucose uptake when compared to healthy cells. Not only was this fundamental component of diabetes maintained in this work, but there was also convincing evidence to support the idea that diabetes imparts a reduced ability of MPCs to stimulate angiogenesis under a variety of contexts.

When considering the effects of MPCs on angiogenesis, it is important to consider the satellite cell life cycle, whereby the majority of satellite cells are quiescent *in vivo*; they are activated with injury, proliferate, and then fuse with existing muscle fibers or with each other (myotube formation) in the process of muscle repair. Interestingly, significant angiogenesis coincides with MPC proliferation in the acute phase of injury. In the current study, conditioned media, or exosomes from proliferating MPCs derived from healthy or diabetic skeletal muscle, was used to treat intact microvessels to gain a better understanding of the impact of disease on satellite cell-induced angiogenesis during this phase of the satellite cell life cycle. Whether isolated from rat or human skeletal muscle, the secretome of the proliferating cells had a reduced ability to stimulate microvascular angiogenesis, using microvascular fragments (MVFs) was evaluated in the present work. Many of the studies evaluating the effect of satellite cell-derived factors on angiogenesis have used human umbilical vein endothelial cells (HUVECs) [19, 43]. Other methods available for assessing the impact of satellite cells on angiogenesis involve the use of outgrowths from skeletal muscle explants, or, like us, the use of MVFs, which are comprised of arterioles, venules, and capillaries [30, 44, 45]. The use of MVFs for evaluating the effects of MPCs and their secretome is appropriate and provides a robust analysis of angiogenesis given that the microvessels are comprised of vascular cells (e.g., endothelial cells, pericytes, resident stem cells) that are involved in microvascular angiogenesis. For example, MVFs have previously been used to demonstrate that satellite cells derived from aged skeletal muscle have a reduced ability to secrete angiogenesis-promoting factors [20], a phenomenon that is consistent with a reduced capillary density in old skeletal muscle [46]. In addition to a reduction in vessel growth, we also observed a down-regulation in gene expression levels of prominent angiogenic markers in MVFs (FLK1, ANGPT-1, and VEGF) (Fig 1).

With the more comprehensive analyses using the rodent cells, we observed the most impactful changes when the entire secretome was used, as compared to experiments where only the exosomes were applied to MVFs. Indeed exosomes have been shown to exert effects over angiogenesis, however, in the current study, the effects were limited. To this end, the majority of the analyses were directed toward gaining a comprehensive understanding of the MPC secretome as a whole, and subsequent analyses were directed toward this objective. Appropriately, an evaluation of the possible differences in the relative amount of angiogenic regulatory factors in the form of cytokines and growth factors, both within the cell and secreted by the cell, was made (Figs 2 and 3). It has been largely established that both the intracellular transcription and regulation of pro- and anti- angiogenic factors and cytokines and their extracellular release, deriving from a multitude of cellular and tissue sources, largely dictate the process of angiogenesis [47–49]. Factors secreted in muscle, including myokines, have been shown to have profound effects on angiogenesis [50, 51]. Among the milieu of identified angiogenic regulating factors, vascular endothelial growth factor (VEGF) is among the most well-studied [52], being known as a prime regulator of angiogenesis and vasculogenesis [53]. It has been studied extensively, especially when examining the mechanisms that regulate the etiology of diseases, such as T2D, that show a distinctive reduction in angiogenesis [11]. Markedly in our studies, VEGF, was the only protein that showed a consistent decrease both intracellularly in Db-MPCs, extracellularly in the Db-MPC secretome, and in the gene expression of MVF treated with Db-MPC CM (Figs 1–3). It should be noted that these results differ from those where Ciaraldi et al. showed no significant difference in the VEGF secreted in control and T2D human myotubes [51]. Similarly, Gavin et al. showed no significant change in VEGF expression between obese and young control human SkM, although a reduction in capillary density was demonstrated [54]. Distinctly, both of these studies look at the effect of mature muscle fibers (Gavin et al.) or myotubes (Ciaraldi et al.), whilst ours explored the role of MPCs, at a non-fully differentiated state, on angiogenesis directly (TE-SkM) and in-directly (CM) using MVF. As shown with our data utilizing TE-SkM, the co-culture of MPCs/MVF had a greater impact at earlier stages (less mature MPCs), giving us further evidence that MPCs, and specifically VEGF, might have a greater influence during muscle regeneration.

Intracellularly, a significant increase in the expression of Leptin in the Db-MPCs was also observed (Fig 2). Leptin has been described as a pro-angiogenic factor for its role in vascular permeability and co-function with other growth factors [55], however, it is also notably upregulated in cases of obesity and T2D [56]. In the Db-MPCs, we also conspicuously see alterations in the inflammatory profiling of the cells, with significant changes in IL-1β, IL-10, and IP-10, a common feature of T2D cell types [57]. Among inflammatory molecules, IL-8 and GROα, have previously been identified as myokines responsible for reduced capillary formation in T2D [19, 43, 51]. In our system, using proliferating MPCs, these changes were not noted, a potential reason being again our focus on the proliferative/regenerative state of MPC/vessel interaction, however, we cannot discount that there may be further growth factors/cytokines involved [58].

Collectively, cytokines and growth factors found within and secreted by the cells may be largely responsible for the changes in angiogenesis that were observed, at least in the context of proliferating MPCs. The influence of other components of the secretome cannot be definitively ruled out. Some of the preliminary work hints at other possible factors that may be involved. For example, a cytokine array (n = 1) containing 29 different rat cytokines and chemokines was performed to determine the relative amounts of immunomodulating agents present in the secretome (S1 File). Expression levels of RANTES, CINC-2, CINC-3, IL-1ra, TIMP1, Fractalkine, VEGF, sICAM-1, IL-6, MIP-1a, and MIP-3a were higher in the control secretome. Only CINC-1 and LIX displayed a more significant content in the diabetic secretome. The majority of the cytokines found in the T2D secretome are involved in the recruitment of myeloid cells, which are important mediators of angiogenesis [59, 60]. A cytokine of particular interest is interleukin-1 receptor agonist (IL-1Ra), given that IL-1Ra expression is reduced in patients with T2D [61].

In order to investigate the interaction between muscle cells and microvessels in a more physiologically relevant context, tissue engineering skeletal muscle (TE-SkM) was used to study the process of MVF sprouting and growth by placing them in direct contact with MPCs harvested from the skeletal muscle of control or diabetic Zucker rats. The goal was to determine whether a decline in MVF growth could be observed in the presence of diabetic MPCs, similar to what was observed in the 2D studies, so that a TE-SkM model *in vitro* that closely resembled the three-dimensional physiological arrangement of myogenic cells and the surrounding vasculature network could be generated. One of the many advantages of TE-SkM is that it allows for the examination of myogenic phenomena in a three-dimensional architecture to better replicate *in vivo* skeletal muscle architecture. To determine if deficits in MPC-induced angiogenesis persisted during myogenic differentiation, MPCs derived from either control or diabetic rats were used to generate TE-SkM containing microvessels. Although the results presented in this study are only preliminary and show a difference only for early time points, it was evident how rat diabetic MPCs could initially slow down the growth of MVFs even when co-cultured with MPCs in a 3D setting. It should be noted, however, that the difference in the growth of MVF was absent after six days of culture, indicating the direct contact between MVF and MPCs may also play a role in regulating the fate of MVF sprouting within the TE-SkM constructs. Regardless, the findings that the diabetic phenotype is largely conserved in myotubes established from diabetic subjects [36, 62, 63], which display impaired pro-angiogenic potential, are in agreement with the findings that were observed in TE-SkM (Fig 6). Based on the findings herein the use of TE-SkM is an approach that can be exploited for further investigation *in vitro* to identify new therapeutic targets that can be strictly regulated to restore a healthy vascular network in diseased diabetic SkM.

Finally, in order to stimulate the acute effects of injury and disease on satellite cell-induced angiogenesis in a 3D arrangement, the secretome taken from injured TE-SkM made from either control or diabetic cells was used to treat microvessels. Deficiencies associated with intercellular communication in skeletal muscle may be most strongly apparent in the context of injury, where the spatiotemporal coordination of separate, but interdependent events (e.g., myogenesis and angiogenesis) need to be finely tuned for successful tissue regeneration and repair. In order to stimulate the acute effects of injury and disease on satellite cell-induced angiogenesis in a 3D arrangement, the secretome taken from injured TE-SkM made from either healthy or diabetic cells was used to treat microvessels. The salient finding from these studies is that MPCs and secretome deriving from a healthy source (control), have an increase in pro-angiogenic factors (VEGF and ANGP-1), while diseased or diabetic MPCs or secretome do not. However, the difference in the ability of the non-injured or injured secretome to influence angiogenesis using MVFs, was not significantly seen, albeit MVFs treated with control injured secretome trended higher than their non-injured counterpart. What was clear, as seen in our other data, when comparing the secretome of control and diabetic MPCs in a proliferative state (in this case induced by injury), the diabetic secretome has a reduced ability to promote angiogenesis. Future work, looking at how different time points between injury and secretome collection, the influence of different magnitudes of injury, or exploration of other factors involved in this interaction are of much interest.

## Conclusions

In summary, in this study, it has been established that the secretome derived from diabetic MPCs have a reduced ability to promote angiogenesis *in vitro* when compared to that of their healthy counterparts. These findings add to the understanding of the communication between satellite cells and microvessels in the context of diabetes and suggest insufficiencies of cells derived from diabetic muscle that contribute to a reduction in microvascular density *in vivo*. Primarily, a downregulation of several angiogenic markers and a decline in the vasculature network of MVF seeded in fibrin were observed upon exposure with secretome derived from MPCs harvested from both rat and human diabetic skeletal muscle sources. Interestingly, the direct comparison between the secretome of control and diabetic rat MPCs revealed some critical differences in the cytokines and growth factor profiles. Finally, a TE-SkM model mimicking the spatiotemporal organization of native skeletal muscle further illustrated the effects of diabetes on angiogenesis, as well as the effect of injury-induced angiogenesis arising from control and diabetic sources. The broad range of analyses included in the current study was useful for adding support to the concept that inadequacies imparted onto resident muscle cells by diabetes contributes to pathology including a reduced vessel density.

## Supporting information

S1 File Supplemental materials and methods: Cytokine dot blot arrays. Analysis of cytokines found in the secretome of both control (+/FA) and diabetic (FA/FA) Zucker rat MPCs. Cytokine Array analysis of the conditioned media derived from both groups tested (n = 1 rat per group).
(DOCX)

## References

[1] Groen BB, Hamer HM, Snijders T, van Kranenburg J, Frijns D, Vink H, et al. Skeletal muscle capillary density and microvascular function are compromised with aging and type 2 diabetes. J Appl Physiol (1985). 2014;116(8):998–1005. doi: 10.1152/japplphysiol.00919.2013 24577061

[2] Garner RT, Weiss JA, Nie Y, Sullivan BP, Kargl CK, Drohan CJ, et al. Effects of obesity and acute resistance exercise on skeletal muscle angiogenic communication pathways. Experimental Physiology. 2022;107(8):906–18. doi: 10.1113/EP090152 35561231

[3] Olfert IM, Baum O, Hellsten Y, Egginton S. Advances and challenges in skeletal muscle angiogenesis. American Journal of Physiology-Heart and Circulatory Physiology. 2016;310(3):H326–H36. doi: 10.1152/ajpheart.00635.2015 26608338 PMC4796623

[4] White TP, Esser KA. Satellite cell and growth factor involvement in skeletal muscle growth. Med Sci Sports Exerc. 1989;21(5 Suppl):S158–63. 2691828

[5] Campion DR. The Muscle Satellite Cell: A Review. In: Bourne GH, Danielli JF, editors. International Review of Cytology. 87: Academic Press; 1984. p. 225–51. 10.1016/s0074-7696(08)62444-4 6370890

[6] Kargl CK, Sullivan BP, Middleton D, York A, Burton LC, Brault JJ, et al. Peroxisome proliferator-activated receptor gamma coactivator 1-alpha overexpression improves angiogenic signalling potential of skeletal muscle-derived extracellular vesicles. Exp Physiol. 2022. 10.1113/EP090874 PMC9949767 36454193

[7] Christov C, Chretien F, Abou-Khalil R, Bassez G, Vallet G, Authier FJ, et al. Muscle satellite cells and endothelial cells: close neighbors and privileged partners. Molecular biology of the cell. 2007;18(4):1397–409. doi: 10.1091/mbc.e06-08-0693 17287398 PMC1838982

[8] Schmalbruch H, Hellhammer U. The number of nuclei in adult rat muscles with special reference to satellite cells. The Anatomical record. 1977;189(2):169–75. doi: 10.1002/ar.1091890204 911042

[9] D’Souza DM, Zhou S, Rebalka IA, MacDonald B, Moradi J, Krause MP, et al. Decreased Satellite Cell Number and Function in Humans and Mice With Type 1 Diabetes Is the Result of Altered Notch Signaling. Diabetes. 2016;65(10):3053–61. doi: 10.2337/db15-1577 27335233

[10] Woo M, Isganaitis E, Cerletti M, Fitzpatrick C, Wagers AJ, Jimenez-Chillaron J, et al. Early life nutrition modulates muscle stem cell number: implications for muscle mass and repair. Stem cells and development. 2011;20(10):1763–9. doi: 10.1089/scd.2010.0349 21247245 PMC3182031

[11] Lash JM, Sherman WM, Hamlin RL. Capillary basement membrane thickness and capillary density in sedentary and trained obese Zucker rats. Diabetes. 1989;38(7):854–60. doi: 10.2337/diab.38.7.854 2661284

[12] Marin P, Andersson B, Krotkiewski M, Bjorntorp P. Muscle fiber composition and capillary density in women and men with NIDDM. Diabetes care. 1994;17(5):382–6. doi: 10.2337/diacare.17.5.382 8062604

[13] Lillioja S, Young AA, Culter CL, Ivy JL, Abbott WG, Zawadzki JK, et al. Skeletal muscle capillary density and fiber type are possible determinants of in vivo insulin resistance in man. The Journal of clinical investigation. 1987;80(2):415–24. doi: 10.1172/JCI113088 3301899 PMC442253

[14] Sheng CY, Son YH, Jang J, Park S-J. In vitro skeletal muscle models for type 2 diabetes. Biophysics Reviews. 2022;3(3):031306. doi: 10.1063/5.0096420 36124295 PMC9478902

[15] Rhoads RP, Johnson RM, Rathbone CR, Liu X, Temm-Grove C, Sheehan SM, et al. Satellite cell-mediated angiogenesis in vitro coincides with a functional hypoxia-inducible factor pathway. American journal of physiology Cell physiology. 2009;296(6):C1321–8. doi: 10.1152/ajpcell.00391.2008 19386789 PMC2692418

[16] Nie Y, Sato Y, Garner RT, Kargl C, Wang C, Kuang S, et al. Skeletal muscle-derived exosomes regulate endothelial cell functions via reactive oxygen species-activated nuclear factor-κB signalling. Exp Physiol. 2019;104(8):1262–73. 31115069 10.1113/EP087396

[17] Levy YA, Ciaraldi TP, Henry RR. Impaired capillary tube formation induced by elevated secretion of IL8 involves altered signaling via the CXCR1/PI3K/MMP2 pathway. Mol Biol Rep. 2021;48(1):601–10. doi: 10.1007/s11033-020-06104-z 33411234

[18] Hettinger ZR, Kargl CK, Shannahan JH, Kuang S, Gavin TP. Extracellular vesicles released from stress-induced prematurely senescent myoblasts impair endothelial function and proliferation. Exp Physiol. 2021;106(10):2083–95. doi: 10.1113/EP089423 34333817

[19] Amir Levy Y, Ciaraldi TP, Mudaliar SR, Phillips SA, Henry RR. Excessive secretion of IL-8 by skeletal muscle in type 2 diabetes impairs tube growth: potential role of PI3K and the Tie2 receptor. American journal of physiology Endocrinology and metabolism. 2015;309(1):E22–34. doi: 10.1152/ajpendo.00513.2014 25944879

[20] Rhoads RP, Flann KL, Cardinal TR, Rathbone CR, Liu X, Allen RE. Satellite cells isolated from aged or dystrophic muscle exhibit a reduced capacity to promote angiogenesis in vitro. Biochemical and biophysical research communications. 2013;440(3):399–404. doi: 10.1016/j.bbrc.2013.09.085 24070607

[21] Colombo M, Raposo G, Thery C. Biogenesis, secretion, and intercellular interactions of exosomes and other extracellular vesicles. Annual review of cell and developmental biology. 2014;30:255–89. doi: 10.1146/annurev-cellbio-101512-122326 25288114

[22] Forterre A, Jalabert A, Chikh K, Pesenti S, Euthine V, Granjon A, et al. Myotube-derived exosomal miRNAs downregulate Sirtuin1 in myoblasts during muscle cell differentiation. Cell cycle (Georgetown, Tex). 2014;13(1):78–89. doi: 10.4161/cc.26808 24196440 PMC3925739

[23] Fry CS, Kirby TJ, Kosmac K, McCarthy JJ, Peterson CA. Myogenic Progenitor Cells Control Extracellular Matrix Production by Fibroblasts during Skeletal Muscle Hypertrophy. Cell stem cell. 2017;20(1):56–69. doi: 10.1016/j.stem.2016.09.010 27840022 PMC5218963

[24] Matsuzaka Y, Tanihata J, Komaki H, Ishiyama A, Oya Y, Ruegg U, et al. Characterization and Functional Analysis of Extracellular Vesicles and Muscle-Abundant miRNAs (miR-1, miR-133a, and miR-206) in C2C12 Myocytes and mdx Mice. PloS one. 2016;11(12):e0167811. doi: 10.1371/journal.pone.0167811 27977725 PMC5158003

[25] Choi JS, Yoon HI, Lee KS, Choi YC, Yang SH, Kim IS, et al. Exosomes from differentiating human skeletal muscle cells trigger myogenesis of stem cells and provide biochemical cues for skeletal muscle regeneration. Journal of controlled release: official journal of the Controlled Release Society. 2016;222:107–15. doi: 10.1016/j.jconrel.2015.12.018 26699421

[26] Acosta FM, Jia UA, Stojkova K, Howland KK, Guda T, Pacelli S, et al. Diabetic Conditions Confer Metabolic and Structural Modifications to Tissue-Engineered Skeletal Muscle. Tissue engineering Part A. 2021;27(9–10):549–60. doi: 10.1089/ten.TEA.2020.0138 32878567 PMC8126424

[27] Acosta FM, Jia UTA, Stojkova K, Pacelli S, Brey EM, Rathbone C. Divergent effects of myogenic differentiation and diabetes on the capacity for muscle precursor cell adipogenic differentiation in a fibrin matrix. Biochemical and Biophysical Research Communications. 2020;526(1):21–8. doi: 10.1016/j.bbrc.2020.03.025 32192775 PMC7328770

[28] Lees SJ, Rathbone CR, Booth FW. Age-associated decrease in muscle precursor cell differentiation. American journal of physiology Cell physiology. 2006;290(2):C609–15. doi: 10.1152/ajpcell.00408.2005 16192302

[29] Stone R 2nd, Rathbone CR. Microvascular Fragment Transplantation Improves Rat Dorsal Skin Flap Survival. Plastic and reconstructive surgery Global open. 2016;4(12):e1140. doi: 10.1097/GOX.0000000000001140 28293502 PMC5222647

[30] Acosta FM, Stojkova K, Brey EM, Rathbone C. A Straightforward Approach to Engineer Vascularized Adipose Tissue Using Microvascular Fragments. Tissue engineering Part A. 2020;26(15–16):905–14. doi: 10.1089/ten.TEA.2019.0345 32070226 PMC7462025

[31] Livak KJ, Schmittgen TD. Analysis of relative gene expression data using real-time quantitative PCR and the 2(-Delta Delta C(T)) Method. Methods. 2001;25(4):402–8. doi: 10.1006/meth.2001.1262 11846609

[32] Acosta FM, Howland KK, Stojkova K, Hernandez E, Brey EM, Rathbone CR. Adipogenic Differentiation Alters Properties of Vascularized Tissue-Engineered Skeletal Muscle. Tissue engineering Part A. 2022;28(1–2):54–68. doi: 10.1089/ten.TEA.2021.0064 34102861 PMC8812504

[33] Strobel HA, Schultz A, Moss SM, Eli R, Hoying JB. Quantifying Vascular Density in Tissue Engineered Constructs Using Machine Learning. Frontiers in Physiology. 2021;12. doi: 10.3389/fphys.2021.650714 33986691 PMC8110917

[34] Mauro A. Satellite cell of skeletal muscle fibers. The Journal of biophysical and biochemical cytology. 1961;9:493–5. doi: 10.1083/jcb.9.2.493 13768451 PMC2225012

[35] Dumont NA, Bentzinger CF, Sincennes MC, Rudnicki MA. Satellite Cells and Skeletal Muscle Regeneration. Comprehensive Physiology. 2015;5(3):1027–59. doi: 10.1002/cphy.c140068 26140708

[36] Gaster M, Petersen I, Hojlund K, Poulsen P, Beck-Nielsen H. The diabetic phenotype is conserved in myotubes established from diabetic subjects: evidence for primary defects in glucose transport and glycogen synthase activity. Diabetes. 2002;51(4):921–7. doi: 10.2337/diabetes.51.4.921 11916908

[37] Gaster M, Rustan AC, Aas V, Beck-Nielsen H. Reduced lipid oxidation in skeletal muscle from type 2 diabetic subjects may be of genetic origin: evidence from cultured myotubes. Diabetes. 2004;53(3):542–8. doi: 10.2337/diabetes.53.3.542 14988236

[38] Fabre O, Breuker C, Amouzou C, Salehzada T, Kitzmann M, Mercier J, et al. Defects in TLR3 expression and RNase L activation lead to decreased MnSOD expression and insulin resistance in muscle cells of obese people. Cell death & disease. 2014;5:e1136. doi: 10.1038/cddis.2014.104 24651439 PMC3973244

[39] Green CJ, Pedersen M, Pedersen BK, Scheele C. Elevated NF-kappaB activation is conserved in human myocytes cultured from obese type 2 diabetic patients and attenuated by AMP-activated protein kinase. Diabetes. 2011;60(11):2810–9. 21911750 10.2337/db11-0263 PMC3198079

[40] Henry RR, Ciaraldi TP, Mudaliar S, Abrams L, Nikoulina SE. Acquired defects of glycogen synthase activity in cultured human skeletal muscle cells: influence of high glucose and insulin levels. Diabetes. 1996;45(4):400–7. doi: 10.2337/diab.45.4.400 8603759

[41] Henry RR, Ciaraldi TP, Abrams-Carter L, Mudaliar S, Park KS, Nikoulina SE. Glycogen synthase activity is reduced in cultured skeletal muscle cells of non-insulin-dependent diabetes mellitus subjects. Biochemical and molecular mechanisms. The Journal of clinical investigation. 1996;98(5):1231–6. doi: 10.1172/JCI118906 8787686 PMC507545

[42] Henry RR, Abrams L, Nikoulina S, Ciaraldi TP. Insulin action and glucose metabolism in nondiabetic control and NIDDM subjects. Comparison using human skeletal muscle cell cultures. Diabetes. 1995;44(8):936–46. doi: 10.2337/diab.44.8.936 7622000

[43] Amir Levy Y, PC T, RM S, AP S, RH R. Adipose tissue from subjects with type 2 diabetes exhibits impaired capillary formation in response to GROα: involvement of MMPs-2 and -9. Adipocyte. 2022;11(1):276–86. 35481427 10.1080/21623945.2022.2070949 PMC9116416

[44] Acosta FM, Stojkova K, Zhang J, Garcia Huitron EI, Jiang JX, Rathbone CR, et al. Engineering Functional Vascularized Beige Adipose Tissue from Microvascular Fragments of Models of Healthy and Type II Diabetes Conditions. Journal of Tissue Engineering. 2022;13:20417314221109337. doi: 10.1177/20417314221109337 35782994 PMC9248044

[45] Hoying JB, Boswell CA, Williams SK. Angiogenic potential of microvessel fragments established in three-dimensional collagen gels. In vitro cellular & developmental biology Animal. 1996;32(7):409–19. doi: 10.1007/BF02723003 8856341

[46] Landers-Ramos RQ, Prior SJ. The Microvasculature and Skeletal Muscle Health in Aging. Exerc Sport Sci Rev. 2018;46(3):172–9. 29652695 10.1249/JES.0000000000000151 PMC6005745

[47] Ucuzian AA, Gassman AA, East AT, Greisler HP. Molecular mediators of angiogenesis. J Burn Care Res. 2010;31(1):158–75. doi: 10.1097/BCR.0b013e3181c7ed82 20061852 PMC2818794

[48] Finn NA, Searles CD. Intracellular and Extracellular miRNAs in Regulation of Angiogenesis Signaling. Curr Angiogenes. 2012;4(102):299–307. doi: 10.2174/2211552811201040299 23914347 PMC3729401

[49] Todorova D, Simoncini S, Lacroix R, Sabatier F, Dignat-George F. Extracellular Vesicles in Angiogenesis. Circ Res. 2017;120(10):1658–73. doi: 10.1161/CIRCRESAHA.117.309681 28495996 PMC5426696

[50] Eckel J. Myokines in metabolic homeostasis and diabetes. Diabetologia. 2019;62(9):1523–8. doi: 10.1007/s00125-019-4927-9 31263909

[51] Ciaraldi TP, Ryan AJ, Mudaliar SR, Henry RR. Altered Myokine Secretion Is an Intrinsic Property of Skeletal Muscle in Type 2 Diabetes. PloS one. 2016;11(7):e0158209. doi: 10.1371/journal.pone.0158209 27453994 PMC4959771

[52] Gerhardt H, Golding M, Fruttiger M, Ruhrberg C, Lundkvist A, Abramsson A, et al. VEGF guides angiogenic sprouting utilizing endothelial tip cell filopodia. J Cell Biol. 2003;161(6):1163–77. doi: 10.1083/jcb.200302047 12810700 PMC2172999

[53] Neufeld G, Cohen T, Gengrinovitch S, Poltorak Z. Vascular endothelial growth factor (VEGF) and its receptors. FASEB J. 1999;13(1):9–22. 9872925

[54] Gavin TP, Stallings HW 3rd, Zwetsloot KA, Westerkamp LM, Ryan NA, Moore RA, et al. Lower capillary density but no difference in VEGF expression in obese vs. lean young skeletal muscle in humans. J Appl Physiol (1985). 2005;98(1):315–21. doi: 10.1152/japplphysiol.00353.2004 15298982

[55] Cao R, Brakenhielm E, Wahlestedt C, Thyberg J, Cao Y. Leptin induces vascular permeability and synergistically stimulates angiogenesis with FGF-2 and VEGF. Proceedings of the National Academy of Sciences. 2001;98(11):6390–5. doi: 10.1073/pnas.101564798 11344271 PMC33478

[56] Katsiki N, Mikhailidis DP, Banach M. Leptin, cardiovascular diseases and type 2 diabetes mellitus. Acta Pharmacol Sin. 2018;39(7):1176–88. doi: 10.1038/aps.2018.40 29877321 PMC6289384

[57] Calle MC, Fernandez ML. Inflammation and type 2 diabetes. Diabetes Metab. 2012;38(3):183–91. doi: 10.1016/j.diabet.2011.11.006 22252015

[58] Latroche C, Weiss-Gayet M, Muller L, Gitiaux C, Leblanc P, Liot S, et al. Coupling between Myogenesis and Angiogenesis during Skeletal Muscle Regeneration Is Stimulated by Restorative Macrophages. Stem Cell Reports. 2017;9(6):2018–33. doi: 10.1016/j.stemcr.2017.10.027 29198825 PMC5785732

[59] Dimberg A. Chemokines in angiogenesis. Curr Top Microbiol Immunol. 2010;341:59–80. doi: 10.1007/82_2010_21 20373091

[60] Rubina KA, Semina EV, Tkachuk VA. Guidance molecules and chemokines in angiogenesis and vascular remodeling. Journal of Evolutionary Biochemistry and Physiology. 2017;53(5):349–67.

[61] Larsen CM, Faulenbach M, Vaag A, Ehses JA, Donath MY, Mandrup-Poulsen T. Sustained effects of interleukin-1 receptor antagonist treatment in type 2 diabetes. Diabetes Care. 2009;32(9):1663–8. doi: 10.2337/dc09-0533 19542207 PMC2732140

[62] Gaster M. The diabetic phenotype is preserved in myotubes established from type 2 diabetic subjects: a critical appraisal. APMIS. 2019;127(1):3–26. doi: 10.1111/apm.12908 30549138

[63] Gaster M. Reduced lipid oxidation in myotubes established from obese and type 2 diabetic subjects. Biochem Biophys Res Commun. 2009;382(4):766–70. doi: 10.1016/j.bbrc.2009.03.102 19324004

