# Supplementary material for: Diabetes diminishes muscle precursor cell-mediated microvascular angiogenesis
Source: PLoS One. 2023 Aug 4;18(8):e0289477. doi: 10.1371/journal.pone.0289477 (PMC10403078; doi:10.1371/journal.pone.0289477)
Supplement: S1 File — Analysis of cytokines found in the secretome of both control (+/FA) and diabetic (FA/FA) Zucker rat MPCs. Cytokine Array analysis of the conditioned media derived from both groups tested (n = 1 rat per group). (DOCX) [file pone.0289477.s001.docx]

**Supplemental File**

**Diabetes diminishes muscle precursor cell-mediated microvascular angiogenesis**

Francisca M Acosta Ph.D.^1,2,#a^, Settimio Pacelli Ph.D.^1,#b^, Christopher R Rathbone Ph.D.^1,2,3,*^

^1^ Department of Biomedical and Chemical Engineering, University of Texas at San Antonio, San Antonio, TX, United States

^2^ UTSA-UTHSCSA Joint Graduate Program in Biomedical Engineering, San Antonio, TX, United States

^3^ Institute of Regenerative Medicine, University of Texas at San Antonio, San Antonio, TX, United States

^#a^ Current Address: Department of Biochemistry and Structural Biology, University of Texas Health Science Center, San Antonio, TX, United States

^#b^ Current Address: Department of Biomedical Engineering, Illinois Institute of Technology, Chicago, IL, United States

**Contents:**

- Supplemental Materials and Methods
- Supplemental Figures

**Supplemental Materials and Methods:**

***Cytokine Dot Blot Arrays***

The expression of 29 different rat cytokines and chemokines were quantified using the Proteome profiler Rat Cytokine Array Panel A (R&D System, Minneapolis, MN) from the conditioned medium derived from MPCs from lean and diabetic conditions. In brief, the culture supernatant was diluted and mixed with a cocktail of biotinylated detection antibodies. The solution was then placed on top of a membrane and incubated overnight. Each growth factor had two identical spots on the membrane, which allowed the detection of each protein in duplicate. Protein detection antibodies bound to the capture antibody were detected using Streptavidin-HRP and chemiluminescent detection reagents. Spot densitometry was performed using a LI-COR Odyssey FC system (LI-COR, Lincoln, NE). Background staining and spot size were analyzed, and results were expressed as relative to control spots.

**Supplemental Figure:**

**
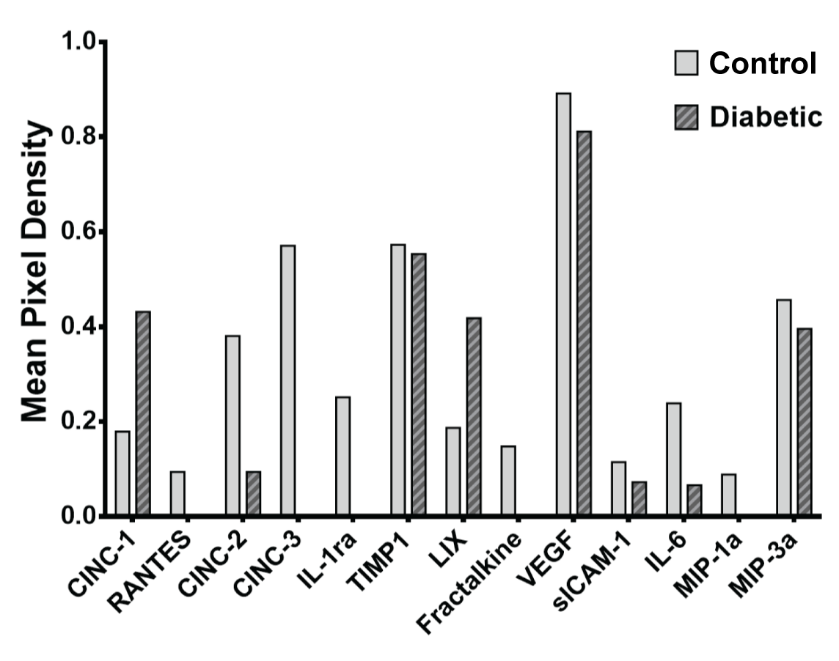
**

**Supplemental Figure 1.** Analysis of cytokines found in the secretome of both control (+/FA) and diabetic (FA/FA) Zucker rat MPCs. Cytokine Array analysis of the conditioned media derived from both groups tested (n=1 rat per group).
